# A Course of Clinical Lectures on Some Important Points of Surgery

**Published:** 1844-11-02

**Authors:** Benjamin C. Brodie


### CLINICAL LECTURES AND REPORTS.

### A COURSE OF CLINICAL LECTURES ON SOME IMPORTANT POINTS OF SURGERY,

*Delivered at St. George's Hospital,*

BY SIR BENJAMIN C. BRODIE.

### SOME DISEASES OF THE BREAST.

Although after thirty years' active service, I have resigned my office as surgeon in this hospital, I shall always feel the deepest interest in its success, and that of the school connected with it, and shall feel happy when I can serve either the institution or the pupils connected with it. I shall always think you have a demand on my attention, and my best wishes for your success will always attend you. And that this may not be mere words, I intend continuing my lectures this season; I have also offered to do so through succeeding sessions, which offer has been accepted; but before I go farther, it is necessary I should explain to you what these lectures are to be, as it is evident they cannot be clinical as hitherto; nor must they be such as in any way interfere with the lectures delivered by Mr. Hawkins and Mr. Babington, but this will be easily managed. Not being limited to time, I shall be able to speak at greater length than on ordinary occasions: again, I shall not be called upon to lecture on any particular disease, so that I shall take up the same disease in different ways, sometimes confining myself to mere symptoms: and although this may appear strange to the scientific pathologist, it will, I am sure, be serviceable to the wants of the students. I shall also endeavour to make up for what is necessarily wanting in the wards of an hospital to complete the practitioner; for it is impossible here to learn all that is wanting for private practice. Persons in different classes of society require different modes of treatment; their constitutions differ; and although you may learn all that is necessary for the treatment of the lower orders, you do not learn the diseases of those in affluence. When I commenced practice, I was often greatly at a loss from this cause. I shall, therefore, call your attention to such diseases as are most frequently met with in the higher classes of society.

In the present lecture, I shall make some observations on the diseases of the breast, no very clear description having been given of them, although of common occurrence. The disease to which I shall particularly refer to-day is one of considerable interest; especially so, because it is quite different from carcinoma, with which it has been frequently confounded. It is not met with in hospital practice, but very often shows itself in private life, and unless I had had the advantage of seeing a large number of private patients, I should not have been able to make out its symptoms and history, as I believe I now can. You will, perhaps, be consulted by a female who has a hard tumour formed in the breast, about the size of a walnut, and moveable; it contains fluid, and you puncture it with a grooved needle, and serum escapes. If the opening be large enough the whole escapes, and nothing remains but the bag or cyst in which it was contained. This fluid varies in colour, being sometimes yellow or straw coloured, sometimes greenish, and at other times of a dark brown. I call it serum because it entirely coagulates by heat, or by the addition of nitric acid; it is, therefore, doubtless serum, with some colouring matter. Sometimes you will find only one of these tumours, whilst at other times there will be several. When this is the case there is generally one large one, and several very small ones; but sometimes the whole breast is filled with them; they frequently occur as large as marbles, but I have seen them as big as an orange. When punctured in the early stage of the disease, there will be no perceptible remains of the tumour afterwards, which proves the cyst to be very thin. It occurs chiefly in single women, or those who have born, no children, but there are exceptions to this rule. It is attended with no pain, except the nervous pain caused by anxiety, and by the attention being constantly directed to the part; it does not effect the axillary glands, and the disease may remain unaltered for several years. Very frequently these tumours form without the patient being conscious of their presence, till accidentally she feels her breast, and finds something like a marble there. In one case of this kind, Mr. Johnson and myself examined the parts carefully, and found several glandular cysts containing nothing but serum. I have said the serum came from cysts, but my belief is that they are only enlargments of the lactiferous tubes; my reasons for coming to this conclusion are these: persons affected by this disease not unfrequently have a discharge of serum from the nipples; and if you press them, you may squeeze out the whole of the serum, so that nothing apparently remains. In one case of this kind, which ultimately I removed by operation, I passed a bristle down one of the tubes, and it went directly into one of the cysts. What I have now described may be looked upon as the first stage of the disease. But although during the early stages of the disease, these cysts contain nothing but fluid serum, if they are allowed to go on, solid matter will be formed in them, and so long as they are neglected this solidity will continue to increase. Where there are several cysts of this kind, you will occasionally find the whole of the breast becomes solid, excepting a few small portions which appear to remain unaffected.

A question very naturally arises here, as to what change in the part is this alteration of structure to be attributed? A lady consulted me who had one of these tumours in her breast, about the size of a walnut; I punctured it with a needle first, and finding it contained serum, I laid it open with a lancet; a large quantity of fluid escaped. I then dressed it with lint to the bottom, meaning to bring on inflammation; a good deal of suppuration followed, and the wound was two months before it healed, and then the disease was apparently quite eradicated. About a year after this, the patient came to me again, and I found, where I had opened the cyst a fungous tumour as large as the cyst I had previously opened. I recommended her to have the breast amputated; the operation was performed, and we found it to be entirely made up of cysts containing fluid matter, and one of a large size, as represented in the drawing on the table. From the inner surface of this cyst there projected a solid tumour, which appeared to be made up of numerous folds, giving it a plicated appearance, covered by membranes continuous with that lining the cyst; and when cut into, it looked like very slightly organised fibrine. You have had recently an opportunity of seeing, in the hospital, a case of this kind; I allude to the woman whose breast was amputated, a short time since, by Mr. Keate. When she first came to the hospital, there was a small tumour about the size of a walnut; it was punctured by Mr. Cutler, and found to contain serum; but within a fortnight it regained its former size. She was then admitted as an in-patient under Mr. Keate, who again punctured it, and let out a dark coloured fluid. On the 21st of December, it was found not only to have filled again, but to have considerably increased in size; it was then laid open with a lancet, and a very large quantity of matter escaped; but in a few days, there was found, projecting from its inner surface, a large fibrous excrescence, partly filling up the cavity of the cyst. It was, therefore, deemed advisable to remove the whole of the breast, which, as you are aware, was done early in January. This is the tumour which was then removed (shewing the preparation); some of the excrescences are pendulous from narrow necks; others have broad bases; some look like recently effused albumine; others, like slightly organised fibrine; some are like fatty tumours; and, occasionally, they put on the appearance of *medullary* disease; not that I think they are of this nature, because many other tumours present a similar aspect. Very many years ago, I had the opportunity of witnessing a similar operation performed by Mr. Freeman, of Spring Gardens; and in my written notes of this case, I find I described it as looking like slightly organised fibrine. In this instance, the tumour occupied one-third of the cavity of the cyst. But this is not the only situation in which these solid masses are formed; you will sometimes find them outside of the cyst, and these will increase in size, till all appear to be united in one solid mass; but if you carefully examine them in this state, you will find them outside, and perfectly distinct from the inner surface of the cyst. Here is another tumour which I removed from a private patient; it is of a similar character, and the breast, in this instance, weighed between seven and eight pounds. On cutting into it, a cavity was found, holding a large quantity of serum; here are, also, other preparations on the table, of a similar character. I said, in this particular instance, the breast weighed upwards of seven pounds, and I have seen other cases where they have attained a similar magnitude. The skin does not always ulcerate in these cases; but, occasionally, it is so distended that it bursts; a large quantity of fluid matter will be discharged, and an ulcer of an unhealthy character will be left, which, if not speedily removed, will wear out the constitution, and destroy the life of the patient. This disease has been much confounded with carcinoma. Some years ago, a very celebrated pathologist showed me a case of this kind, and his opinion was that it was carcinoma; and I believe, in many cases of this kind, where amputation was performed for cancer, it has been nothing more than this disease, and not in any way of a malignant character. It is true a patient may be destroyed by these tumours; but this is no reason why it should be malignant. It is a local affection, and does not spring from any evil of constitution; and when the part is amputated, the disease never returns. The private patient from whom I took the preparation on the table, lived many years after the operation, and had no return of the disease. Another lady, from whom I removed a similar tumour six years ago, is now alive and well. Sir E. Home thought the disease was malignant, and always advised amputation; and I recollect that a patient, on whom he operated, I saw twenty years afterwards, and she was perfectly well. I am therefore justified in saying the disease is not malignant.

In the early stage of the disease it may sometimes be cured without operation, as in the following case:—A lady came to me some twenty years ago, with a tumour of this description, and as I did not understand the disease so well then as I do now, I advised her to have it removed. She consented, and the day was fixed for the operation; but as something occurred which prevented its being done then, I said we would try an experiment, and ordered her a stimulating embrocation, and, in a short time, it had entirely disappeared. The same thing occurred a second time, and in neither case was there any return. From the good effect which followed its application in these two cases, I was led to try it in several others, and it has repeatedly been of great service. Mr. Cutler tried it with the woman now in the hospital, and I suspect its failure is to be attributed to its not being applied properly, or for a sufficient length of time. I dare say I have entirely succeeded in ten or twelve instances. The best application for the purpose is, I think, the following:—

℞ Spiritus vini rect.

Sp. camphoræ, aa ℥iiss.

Liq. plumbi subacet. ℥j M.

I believe this form was originally employed by Mr. Pott for other purposes; but as these proportions seem to answer very well, I have never altered them. Flannel is to be soaked in this, and applied over the whole of the part, taking care to wet it with the embrocation seven or eight times in the course of the twenty-four hours. At first, it merely deposits the oxide of lead on the skin; but in a short time the part becomes tender, and if its application be persevered in, the part will become excessively tender and blistered; the patient should then leave it off a few days, and resume it as soon as the parts have a little recovered. In this way, it must be persevered in for several weeks, or even months; but, generally, if it is serviceable, it will be evident when it has been applied six weeks or two months, but it must be persevered with; when the skin has been once stimulated to any extent, it is easily acted upon a second time, so that perhaps the patient will not be able to bear it more than two days in succession. In some cases I tried blistering, keeping the surface open with savine ointment for a fortnight, and then healing it; repeating this for several successive times, and I think this answered best, but it is more troublesome. I have also employed a strong solution of iodine, but it did not answer so well as the embrocation. When using the embrocation, I have sometimes punctured these tumours, and perhaps this may have assisted in their removal; but my own opinion is, that it did not. When I have examined these tumours, after puncturing them, I have found something like a little kernel, which, I suppose, is the collapsed cyst. If the disease is in an advanced state, stimulants will do no good. Nothing, then, has any power over it, so that its removal by operation becomes indispensable. This disease, as I have said before, is not cancerous; but still it should be removed; because, if allowed to remain, the local irritation will destroy the life of the patient; and, if removed, it will not return. If you operate at all, you must remove the whole of the breast, for it is no use taking away small portions. It is better to perform the operation whilst the tumour is small; nevertheless, you are not to be deterred by its magnitude, because it is not in this disease as in carcinoma: there is, in fact, no danger: and I have seen a great many cases where the operation has been performed, and the disease has never returned, There is one observation, in particular, which you must not forget, viz.: that in amputating the breast, great care should be taken to remove it entirely; it is very possible to leave small portions remaining—I am sure I have done it myself—and although no particular harm results from it, still it may render a second operation necessary. In a patient operated on by Sir E. Home, there occurred small cysts about four years afterwards; these I believe to have been left in the operation, being at that time imperfectly formed, and very small. The preparations on the table will illustrate many of the points on which I have been speaking; and a knowledge of this disease will be of great advantage to you, as it will enable you to distinguish a not very uncommon form of mammary disease from scirrhus, and sometimes to quiet the fears of your patient, without resorting to an operation.—*London Med. Times.*

